# Effect and Analysis of Bacterial Lysates for the Treatment of Recurrent Urinary Tract Infections in Adults

**DOI:** 10.3390/pathogens9020102

**Published:** 2020-02-06

**Authors:** Ricardo E. Ahumada-Cota, Ulises Hernandez-Chiñas, Feliciano Milián-Suazo, María E. Chávez-Berrocal, Armando Navarro-Ocaña, Daniel Martínez-Gómez, Genaro Patiño-López, Erika P. Salazar-Jiménez, Carlos A. Eslava

**Affiliations:** 1 Doctorado en Ciencias Biológicas, Universidad Autónoma de Querétaro, 76230 Querétaro, Mexico; ricardoeahumada@gmail.com; 2 Laboratorio de Patogenicidad Bacteriana, Unidad de Hemato-Oncología e Investigación Hospital Infantil de México Federico Gómez/División de Investigación, Facultad de Medicina, Universidad Nacional Autónoma de Mexico, 06720 Ciudad de México, Mexico; malenachavezb@yahoo.com.mx (M.E.C.-B.); paloma9978@hotmail.com (E.P.S.-J.); 3 Departamento de Salud Pública, Facultad de Medicina, Universidad Nacional Autónoma de México, 04510 Ciudad de Mexico, Mexico; arnava@unam.mx; 4 Facultad de Ciencias Naturales-Universidad Autónoma de Querétaro, 76230 Querétaro, Mexico; feliciano.milian@uaq.mx; 5 Laboratorio de Biología Molecular, Departamento de Producción Agrícola y Animal, Universidad Autónoma Metropolitana Xochimilco, 04960 Ciudad de México, Mexico; dmartinez@correo.xoc.uam.mx; 6 Laboratorio de Investigación en Inmunología y Proteómica, Sección de Biología Celular de Linfocitos Unidad de Hemato-Oncología e Investigación Hospital Infantil de México, Federico Gómez, 06720 Ciudad de México, Mexico; gpatino@himfg.edu.mx

**Keywords:** uropathogenic *E. coli*, recurrent UTI, bacterial lysates

## Abstract

Urinary tract infection (UTI) is a relevant public health problem, economically and socially affecting the lives of patients. The increase of antimicrobial bacterial resistance significantly hinders the treatment of UTIs, raising the need to search for alternative therapies. Bacterial lysates (BL) obtained from *Escherichia coli* and other pathogens have been used to treat different infectious diseases with promising results. This work aims to evaluate the effect and composition of an autologous BL for the treatment and control of recurrent UTIs in adults. The results show remission in 70% of the patients within the first three months after the administration of BL, while the infection is maintained under control for 6–12 months. The analysis by liquid chromatography–mass spectrometry (LC-MS) of the BL fractions recognized by the sera of patients shows the presence of cytosolic proteins, fimbriae, OMPs, and LPS. Our study demonstrates that the autologous BL contributed to the treatment and control of recurrent UTIs in adults, and its composition shows that different surface components of *E. coli* are potential immunogens that could be used to create a polyvalent protective vaccine.

## 1. Introduction

Urinary tract infection (UTIs) is an important health concern worldwide, with over 150 million cases reported every year [1]. The most common bacteria causing UTIs are Gram-negative bacteria, mainly strains belonging to uropathogenic *E. coli* (UPEC) pathotype [1,2,3]. Antibiotics are the most commonly prescribed therapy to treat UTIs; however, multidrug-resistant (MDR) UPEC strains have recently emerged, thus hampering the treatment and control of these infections [4,5]. Accordingly, the WHO has included *E. coli* in a list of pathogens for special consideration due to their MDR profile and the difficulty this poses for the treatment and control of the illnesses these bacteria give rise to. It has also encouraged the development of alternative therapies for the treatment of these diseases [6]. In Mexico, UTIs are the third cause of morbidity among the general population and the second in women [7]. Moreover, it is known that persistent UTIs are common and the illness becomes recurrent in most cases [8]. Aimed at the prevention and control of recurrent UTIs, SolcoUrovac, OM-89/Urovaxom, and most recently Uromune^®^, are bacterial immunostimulants manufactured with a mixture of whole bacterial lysates (BL) from several uropathogen strains that have shown variation in their efficacy [4,9,10]. Unfortunately, the assembly and composition of these BLs are unknown and thus limit the capacity for a deep understanding of the BL mechanism. Here, a multicentric prospective study was performed with adult patients from private practices diagnosed with recurrent UTIs treated with an autologous BL. Additionally, the immunogenic components of the BL from the four most prevalent *E. coli* serogroups were identified, gaining insight that will contribute to the development of a vaccine (polyvalent immunogenic stimulant) to treat and control UTIs.

## 2. Results

### 2.1. Efficacy of the Autologous BL 

#### 2.1.1. Patients 

From April 2014 to November 2018, 22 women and 3 men between the ages of 19 and 84 years, diagnosed with recurrent UTIs, were recruited for this study. All patients presented symptomatic bacteriuria at the beginning of the study and started to receive treatment with an autologous BL. Only 13 patients remained for the completion of the one-year follow-up, while some patients stayed for 33 months (Table S1).

#### 2.1.2. UTIs Etiology 

After analyzing 181 urocultures from 25 patients, 111 (61.3%) had a count of ≥10^5^ CFU/mL. From the 1110 colonies recovered, the most frequent bacteria were members of the Enterobacteriales family (84.7%), where *E. coli* (64.8%) was the principal causative agent, followed by *Proteus* spp. (11.7%), *Klebsiella* spp. (4.5%), *Citrobacter* spp. (3.6%), and unidentified Gram-positive cocci (15.3%). A single bacterial strain was presented by 98% of the urocultures, while mixed cultures of *E. coli* and *Proteus* spp. were present in three urine samples. Serotyping of the 720 isolates indicated that 645 (89.6%) belonged to different, recognized *E. coli* serotypes and 75 were non-typable; 16 different serotypes were identified, seven of them representing 78.4% of all strains (O25: H4, O75: HNM, O6: H1, O8: HNM, O20: H9, O22: H1, O44: HNM) (Table 1).

#### 2.1.3. Effect of the Autologous BL in Patients with Recurrent UTI

Analysis of the autologous BLs included the 13 patients that completed ≥ 1 year in the study. At the beginning of the protocol all patients presented symptomatic bacteriuria (10^5^ CFU/mL); *E. coli* was isolated in 91% (12/13) and *Proteus* spp. in 8% (1/13) of patients. In the first three months of treatment with the autologous BL, UTIs were eliminated in 69% (9/13) of patients, with a lapse of 3–4 months with no further infection. After this period, patients presented reinfection caused by a different strain. An autologous BL was manufactured with the new strain and most cases showed the resolution and control of the UTI (Table S1). Only 31% (4/13) of the patients presented a persistent UTI that could not be resolved and was associated with O25: H4 and O75: HNM serotypes.

### 2.2. Immunoassays using the sera from pre- and post-treatment patients with BL and donors with no previous UTIs 

#### 2.2.1. ELISA and Western-Blot of the BLs 

**Figure 1:**
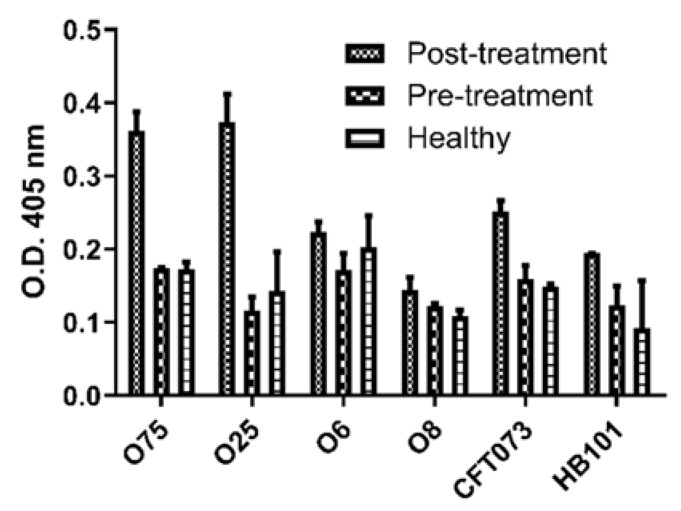
Sera reactivity to the BLs. ELISA assay for the evaluation of the sera reactivity of patients pre- and post-treatment and donors with no UTIs (Healthy) to the 4 BLs of the most frequent isolated serogroups (O75, O25, O6, and O8) and control strains (CFT073 and HB101).

To evaluate the reactivity of the sera from patients before (pre) and after (post) the treatment with the autologous BL and donors with no previous UTIs, an ELISA assay was performed using the four most prevalent serogroups (O75, O25, O6, and O8) isolated from the urocultures (Table 1). The analysis showed higher reactivity of the post-treatment sera to the BL of O75, O25, and CFT073 (serogroup O6) strains than of the sera from pre-treatment and donors (*p* < 0.05) (Figure 1).

**Figure 2:**
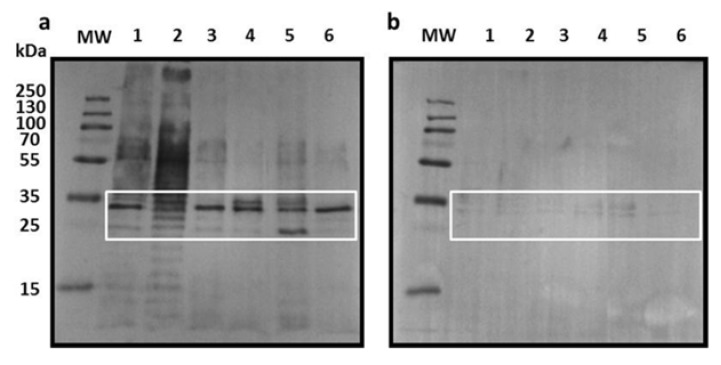
Sera reactivity to BL fractions. Western-Blot analysis of the BLs fractions resolved in a 12% SDS-PAGE, transferred to a PVDF membrane and incubated with the sera of patients (**a**) post-treatment and (**b**) donors with no UTIs. MW: molecular weight marker; lanes 1-6: O75 BL, O25 BL, O6 BL, O8 BL, CFT073 BL, and HB101 BL, respectively. Highly immunogenic fractions with post-treatment and donor sera are inside the white square.

After this, to identify the immunogenic proteins present in the BL, a Western blot analysis using the same BLs (O75, O25, O6, O8, CFT073, and HB101) was performed. Two protein fractions (~25 and ~35 kDa) were highly reactive with the post-treatment pooled sera (Figure 2a), but were weakly reactive using those of pre-treatment patients and donors with no UTIs (Figure 2b). Additionally, the post-treatment pooled sera showed reaction with a ~55 kDa fraction and the presence of LPS in the BL from O75 and O25 strains (Figure 2a), which was not seen in the donors with no UTIs pooled sera.

#### 2.2.2. Identification of Immunogenic Proteins

**Figure 3:**
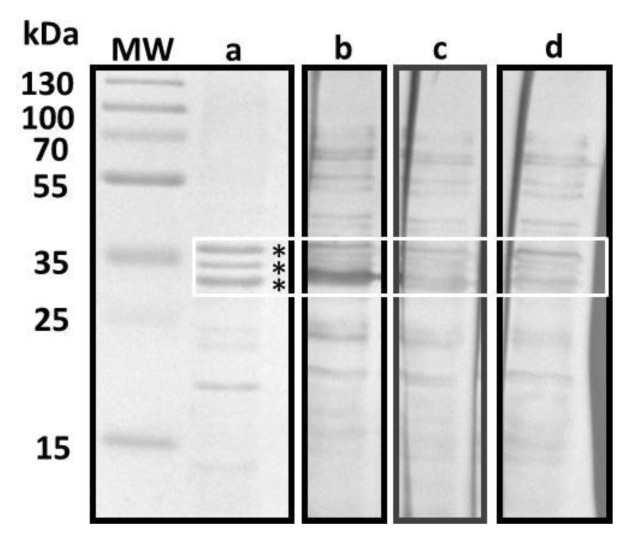
Western blot test. Sera reactivity to OMPs. The OMP enriched extract from (**a**) *E. coli* O75 was resolved in a 12% SDS-PAGE stained with Coomassie blue. Proteins were transferred to a PVDF membrane and incubated with the sera of (**b**) post and (**c**) pre-treatment patients and (**d**) donors with no UTIs. OMPs between 30 and 37 kDa are inside the white square. Protein fractions marked with an asterisk were sent for sequencing and are listed in Table S2.

To identify the immunogenic proteins detected in the BLs, two different methods were employed. The first method was performed to enrich the recovery of outer membrane proteins (OMPs) in the *E. coli* O75 strain (Figure 3). The SDS-PAGE analysis showed the presence of proteins between 10 and 55 kDa (Figure 3a); however, the Western blot analysis revealed a reactivity to proteins ranging from 10 to 70 kDa in the three different sera utilized (Figure 3b–d). As in the Western blot analysis of the BLs, the highest reactivity was observed in the sera from post-treatment patients to proteins between 30 and 37 kDa (Figure 3b).

**Figure 4:**
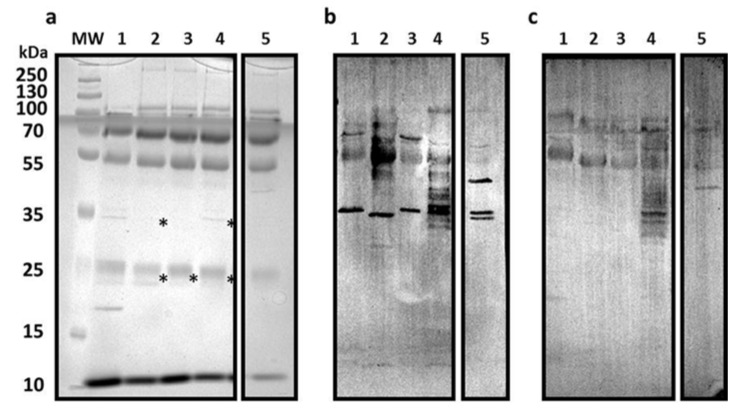
Western blot test. Sera reactivity to *E. coli* surface structures. The bacterial surface structures extracts were resolved in (**a**) 12% SDS-PAGE stained with Coomassie blue. The proteins were transferred to a PVDF membrane and incubated with the sera of (**b**) post-treatment patients and (**c**) donors with no UTIs. MW: molecular weight marker; lanes 1–5: CFT073 BL, O75 BL, O6 BL, O8 BL, and HB101 BL, respectively. Protein fractions marked with an asterisk were sent for sequencing and are listed in Table S2.

The second method was employed for the overexpression and recovery of bacterial surface structures in the *E. coli* O75, O6, O8, CFT073, and HB101 strains. The SDS-PAGE analysis showed the presence of protein fractions between 10 and 250 kDa, and the immunogenic fractions in the range from 55 to 100 kDa (Figure 4a) revealed by the Western blot analysis. The major reactivity was observed in the sera from post-treatment patients to proteins between 33 and 35 kDa (Figure 4b). Interestingly, the sera of the donors with no UTIs detected the presence of O8 LPS (Figure 4b,c).

Finally, the identification of the immunogenic fractions was performed via LC-MS. The analysis showed the presence of cytosolic proteins Udp, MasZ, and GpmA, bacterial surface proteins BamB and BamC, part of the Bam complex, fimbrial adhesin FimH, and OmpC porin. Remarkably, the OmpA protein showed the highest immunogenic reactivity with the sera from post-treatment patients.

### 2.3. ompA Relevance in Different E. Coli Pathotypes

The PCR assay for the evaluation of the presence of *ompA* showed a prevalence of 55% (81/148) in all *E. coli* strains (Table 2). Moreover, the analysis revealed no difference between the presence of this gene in different *E. coli* pathotypes; however, all strains used for the immunoassays were *ompA* positive. Additionally, to confirm that there was no correlation between the presence of *ompA* and the *E. coli* pathotypes, a phylogenetic analysis was performed using 950 sequences of this gene from the GenBank. The analysis revealed that *ompA* was highly conserved with >97% similarity and no group between the 6 UPEC GeneBank genomes was formed (Figure S1).

## 3. Discussion

UTIs are an important health concern worldwide and their treatment has recently become extremely difficult due to antimicrobial resistance [11]. Women are particularly affected by UTIs, with up to 40% of them having at least one episode in their lifetime [12]. The prevalence of UTIs in Mexico is higher among ≥ 18 year-old women, making these illnesses a relevant problem, observed from clinical and epidemiological perspectives [7]. Conditions of different natures, such as urinary tract dysfunction and genetic mechanism involved in the innate immune response to infections, are involved in the development of recurrent UTIs [13]. Due to the increase in multidrug-resistant bacteria, the WHO released a list of important pathogens where *E. coli* has been included among those in critical priority [6]. This has encouraged the development of alternative therapies for the treatment and control of several infections, among these, UTIs are relevant because of their clinical and social impact, especially in women. In this study, the efficacy of an autologous BL for the treatment and prevention of UTI was analyzed. Overall, 92% of patients were women; all patients presented a recurrent UTI (some for many years) and a deficient response to antibiotic treatment. In accordance with similar studies, the most frequently isolated pathogen was *E. coli* (65%) and the antimicrobial susceptibility tests of these strains showed multidrug and extensive drug resistance (manuscript in preparation) [1,2,14,15]. To identify whether patients presented a recurrent UTI or not, characterization by typing with a specific sera was performed. In total, 90% of *E. coli* isolated strains were serotyped and the presence of eight classic UPEC serogroups were observed; still, several non-classic serogroups were also identified. Other studies addressing the serotyping of *E. coli* strains associated with UTIs have reported similar results, mentioning classical and non-classical UPEC serogroups. When the results of non-classical UPEC serogroups obtained here were compared, these were different from those reported by studies in Mexico and other countries [14,15,16,17,18,19,20]. Besides showing the differences between serogroups around the globe, these statements confirm the fact that certain strains, grouped by Clermont [21] in the *E. coli* commensal group, are involved in the reinfections of patients with UTIs [22].

Autologous BLs are manufactured with the isolated bacteria from the culture of the infected site, inactivated by heat, and homogenized in a suspension, favoring IgG and IgM production and T-lymphocyte activation [23,24]. The autologous BL treatment observed in this study involved not only the disappearance of symptoms, but also the absence of infection for up to four months—a condition not observed with the traditional antimicrobial treatment. These products have been used to solve infections that are difficult to treat and are defined as biological medicines displaying the immunogenic components of the microorganism without synthetic chemical compounds via a natural and normal physiological route that induces an active protective immunization [25]. Here, the mucosal immune system is of great importance due to the ongoing exposure this system has to several and abundant antigens, which is the reason why a regulated immunological reaction is activated at the local and systemic levels [26]. Different commercial products for the treatment of UTIs have been made. OM-89 manufactured with 18 uropathogen strains has been tested for the control of UTIs; studies have described its role as an innate and adaptive immune response stimulant, activating dendritic and T-regulator cells, favoring activation and cellular migration to the urothelial mucosa [25,27]. Similarly, Uromune^®^ is a more recent commercial product manufactured with equal amounts of strains of *E. coli*, *K. pneumonia*, *P. vulgaris*, and *E. faecalis*, which has also been described as a mucosal immunostimulant [10,28]. In the present study, an autologous BL, applied orally, was capable of stimulating the bladder mucosa. It is known that these types of products activate a systemic immune response, favoring pathogen elimination [29,30,31]. Unfortunately, four patients with UTIs associated with *E. coli* strains O25: H4 and O75: NM did not respond to the treatment with the autologous BL and the UTIs persisted. Several studies have associated UPEC O25: H4 strains to persistent UTIs due to the formation of intracellular bacterial communities [32].

UPEC involvement in UTI pathogenesis is related to the acquisition of several genes associated with mucosal immune response evasion mechanisms and a limitation of humoral immune response by the host [22,29,33]. Analysis of the BL components showed a reaction against LPS, an important component of Gram-negative bacteria defined as a PAMP, which the mammal immune system uses for the detection of bacteria [34,35]. However, the LPS is known to be poorly immunogenic in its pure form [36]; this is the reason why we considered the response in patients to be increased by a protein fraction acting as an immunogenic molecule or adjuvant. To know which bacterial components were present in the BL and were recognized by the IgG of the patients, two methods were employed. The first method was performed for the enrichment of OMPs in the O75 strain, while the other technique favored the expression of superficial structures in the O75, O6, O8 CFT073, and HB101 strains. Curiously, OmpA was the detected protein in the two different methods used in this study. This protein is present in several enterobacteria with different factors involved in its expression [37]; for instance, Mobley et al. reported that UPEC expressed a higher amount of OmpA while being grown in urine [38]. Additionally, OmpA is known to be a major virulence factor due to its contribution to biofilm production, adherence, and intracellular invasion to the epithelial cells of the bladder, hence creating internal bacterial communities [39,40]. The presence of *ompA* in several *E. coli* pathotypes has been reported [41]; therefore, a PCR assay for the detection of this gene was performed in a collection of *E. coli* strains isolated from different sources, and a prevalence of 55% was observed. Similar studies also reported a prevalence of 93.3% in several pathotypes [42]; in Egypt, *E. coli* strains associated with UTIs had a prevalence of 17.2% [43]. These results show that OmpA is not an exclusive factor of a particular *E. coli* pathotype. Several studies show that differences in the sequence of OmpA are related to the capability that some *E. coli* strains have to adhere to and invade cells [44]. To evaluate the distribution and sequence similarity of the *ompA* gene among *E. coli* pathotypes, a database was assembled using the gene sequences from the GenBank. In concordance with the gene prevalence results, the computational analysis revealed that there is no distinctive sequence pattern of *ompA* between the *E. coli* pathotypes. Conversely, Camparabi et al. [45] observed no correlation between the *ompA* sequences of invasive-adherent and commensal *E. coli* strains.

*E. coli* strains associated with UTIs employ outer membrane structures that contribute to the adherence and invasion of the uroepithelial cells, while others protect the bacteria from phagocytosis (endotoxin, capsule, and biofilm). These structures obstruct the innate and adaptive immune response, and consequently, persistent and recurrent UTIs occur in the host. The BL components analysis revealed the presence of cytosolic proteins, fimbriae fractions, LPS, and OMPs. Recognition of the BL fractions by the sera from treated patients and not by the sera from pre-treated patients or healthy donors suggests that thermally treated bacteria expose different epitopes to the mucosal immune system, further activating the systemic response. The results obtained here show the diversity of *E. coli* serogroups associated with UTIs and the efficacy of the treatment with autologous BL for the control of these infections. We showed that OmpA is a highly immunogenic protein and thus a potential candidate for the development of a polyvalent vaccine against recurrent *E. coli* associated UTIs.

## 4. Materials and Methods 

### 4.1. Study Design 

#### 4.1.1. Study Population

To assess the efficacy of the autologous BL, a multicentric prospective study was performed with adult patients from private practice diagnosed with ongoing recurrent (≥3 per year) UTIs and a deficient response to antibiotic treatment. Patients were asked to remain in the study for a year. Some of them were excluded from the study if they showed immune response alterations or had received antibiotic treatment 30 days prior to assessment. Due to the type of study, sampling was consecutive and non-probabilistic. The protocol was approved by the Research and Ethics Committee of the Medicine Faculty at the National Autonomous University of Mexico (project number 055/2016), and patients and healthy donors signed an informed consent research form before the beginning of the study.

#### 4.1.2. Urine Culture

The sampling of urine was performed monthly according to traditional procedures [46]. Each urine sample was used for a urinalysis with a reactive test strip for 10 parameters (Mission, USA) and a bacterial culture. A semi-quantitative bacterial count was performed spreading 100 µl of urine on Luria-Bertani agar (DIBICO, US); for bacterial isolation, urine sediment was streaked on Blood agar (BA) (DIBICO, US) with 5% sheep blood and MacConkey agar (BD BIOXON, Mexico). Ten colonies were randomly selected and recovered from all positive cultures (≥10^5^ CFU/mL) and a Gram stain test was performed. Gram-negative colonies growing in MacConkey agar were further characterized using IMViC biochemical tests.

#### 4.1.3. Sera from Patients and Donors with no UTI

Sera samples were collected from each patient before (pre) and after (post) 2 months of the beginning of the study and donors with no UTIs 6 months before the study. All participants signed an informed consent form and the nature of the study was explained thoroughly to each of them. Three different pools were manufactured: pooled serum from patients before (1) and after the BL treatment (2), and final pooled sera from donors with no UTIs (3). Each pool was manufactured with equal amounts of serum from 4 individuals in each group and were utilized for the ELISA and Western blot assays. Only the sera from patients that stayed at least 1 year in the study was used.

#### 4.1.4. Serotyping of *E. coli* Isolates

Strains identified as *E. coli* were serotyped by agglutination assays using 96-well microtiter plates and rabbit antisera against O1 to O187 somatic (O) antigens and 53 flagellar (H) antigens prepared in rabbits (SERUNAM, registered trademark in Mexico, 323158/2015) using the method described by Orskov and Orskov [47] with minor modifications. The patient was considered to be colonized by the isolated strain when 8 out of 10 isolates belonged to the same serotype.

#### 4.1.5. Autologous BL

The autologous BL was manufactured using the isolated strain from the patient uroculture. In brief, strains were streaked by massive growth on Luria-Bertani agar in 10 assay tubes (16 × 150) and incubated at 37 °C for 18 h; from these cultures, using saline solution (0.85%), a bacterial suspension of 80 mL with a final concentration of 10^8^ CFU/mL was prepared using MacFarland standard 3. The suspension was inactivated by effluent steam (110 °C for 1 h), centrifuged, and sterilized by filtration (0.22 µm). The filtered solution was dosed in 8 vials (10 mL/vial) and a sterility test by incubation (37 °C for 24 h) was done. An oral dose under aseptic conditions of 2 mL/day for a month was indicated to the patient. Afterwards, if the uroculture was negative, two boosters (two additional months) were given to the patient. When the uroculture was positive, a new BL was prepared and administered to the patient with the same specifications.

### 4.2. Components of BLs 

#### 4.2.1. BL Manufacturing

The BL used for the immunodetection assays was manufactured, as described above, with an extra step for concentration by Four *E. coli* strains belonging to the most prevalent serogroups (O75, O25, O6, and O8) isolated from patients were used for the detection of immunogenic components of the BLs; *E. coli* strains CFT073 and HB101 were used as controls. Strains were kept under freezing conditions (–80 °C) before use and were streaked in TSA (BD BIOXON, Chapultepec, Mexico) for activation lyophilization (Labconco, Kansas City, MO, USA).

#### 4.2.2. OMPs Recovery 

To recover the OMPs, the method described by Molloy et al. [48] was performed with minor modifications. In brief, bacterial strains were cultured in 500 mL of Luria broth (DIBICO, US) and incubated with aeration (200 rpm) at 37 °C for 16 h. Afterward, bacterial cells were recovered by centrifugation (8,000× *g* at 4 °C for 10 min), and the pellet was resuspended and washed twice in 10 mM HEPES (pH 7.0). Bacterial cells were lysed by two passages through a French pressure cell (American Instrument Company, Hartland, WI, USA) at 20,000 lb/in^2^ before centrifugation (8000× *g* at 4 °C for 10 min) to remove unbroken cells and cell debris. The supernatant was diluted in 0.1 M sodium carbonate (pH 11) to a final volume of 50 mL and stirred on ice for 1 h. Carbonate-insoluble membranes were collected by centrifugation (112,000× *g* at 4 °C for 1 h), and the pellet was washed with 10 mM HEPES (pH 7.0) and collected by centrifugation (112,000× *g* at 4 °C for 30 min). The final pellet was resuspended in 1 mL of 10 mM HEPES (pH 7.0) and supplemented with 100 U protease inhibitor cocktail (Sigma-Aldrich, Louis, MO, USA).

#### 4.2.3. Bacterial Surface Structures Recovery

To overexpress the bacterial surface structures, the method described by Mazariego-Espinoza et al. [49] was performed with minor modifications. In brief, bacterial strains were streaked by massive growth on TSA supplemented with 5% sheep blood and incubated at 37 °C for 24 h in a 5% CO_2_ atmosphere. Afterwards, bacteria were recovered using PBS and vortexed vigorously for 30 min, before centrifugation at 10,000× *g* at 4 °C for 30 min. The supernatant was collected and centrifuged at 18,000× *g* at 4 °C for 30 min and the new supernatant was centrifuged at 72,000× *g* at 4 °C for 4 h. The final pellet was solubilized in PBS and supplemented with 100 U protease inhibitor cocktail (Sigma-Aldrich, Louis, MO, USA).

#### 4.2.4. Protein Concentration

To standardize the protein amount of BL and protein extracts to use for the different assays, the protein concentration was calculated using a NanoDrop 2000 spectrometer (Thermo Scientific, Waltham, MA, USA).

#### 4.2.5. ELISA

The reactivity of the different pooled sera was evaluated by ELISA assays. In brief, a 96-well microplate (Inmulon™ 1 B, Thermo Scientific, Waltham, MA, USA) was coated with each BL (0.8 µg protein/well) in 100 µl of coating buffer (100 mM Carbonate buffer, pH 9.6). Unspecific binding sites were blocked with 1% bovine serum albumin (BSA) and PBS at 37 °C for 1 h. Different pooled sera were diluted at 1:50 in PBS with 1% BSA and incubated at 37 °C for 1 h. After intensive washing with 1% PBS Tween (PBS-T) buffer, the anti-human IgG peroxidase antibody (Millipore, Darmstadt, Germany) was added and incubated at 37 °C for 1 h. The detection reaction was visualized by incubating the sample with H_2_O_2_-ABTS (Sigma-Aldrich, Louis, MO, USA) substrate chromogen at 37 °C for 20 min.

#### 4.2.6. Western-Blot

To identify the immunoreactive fractions, western-blot assays were performed using the different BLs, OMPs, and bacterial surface structures extracts. In brief, 30 µg of proteins were mixed with Laemmli buffer and heated in a boiling bath (100 °C for 10 min). The proteins were analyzed in an SDS-PAGE (12% solving gel) (Mini PROTEAN^®^, Bio-Rad, Hercules, CA, USA) and transferred to a PVDF membrane (Millipore, Germany) using a Mini Trans-Blot^®^ (Bio-Rad, Hercules, CA, USA). The membranes were blocked overnight with skimmed milk (5%) and washed 3 times with PBS-T (0.05%) before individual incubation with the respective sera (1:100). Afterwards, membranes were washed 3 times with PBS-T (0.05%) before incubation with the anti-human IgG peroxidase antibody (Millipore, Darmstadt, Germany) (1:3500). The reaction was visualized adding 0.01 M Tris-HCl (pH 6.8) in the presence of H_2_O_2_ and 4-chloro-1-naphtol (Sigma-Aldrich, Louis, MO, USA).

#### 4.2.7. LC-MS Protein Identification 

The protein fractions identified as immunogenic in the previous assays were selected for further characterization. These fractions were cut from the SDS-PAGE Coomassie R-250 stained gels and sent for LC-MS identification to the Laboratorio Universitario de Proteómica del Instituto de Biotecnología de la UNAM (Cuernavaca, México). The analysis of the spectrometer data was performed using Proteome Discover 1.4 (Thermo-Fisher, Waltham, MA, USA) software through the Sequest HT search engine. The identity exploration was executed using the inverted Decoy database UNIPROT for *E. coli*, with an FDR-False Discovery Rate (Minimum) of 0.01 and FDR 0.05 (Maximum).

#### 4.2.8. PCR for the Detection of *ompA* in *E. coli* Strains 

To determine the frequency of the *ompA* gene in different *E. coli* pathotypes, a PCR assay was performed using the forward 5′-AGCTATCGCGATTGCAGTG-3′ and reverse 5′-GGTGTTGCCAGTAACCGG-3′ primers previously described by Ewers et al. [50]. *E. coli* characterized strains (198) from the Laboratorio de Patogenicidad Bacteriana del Hospital Infantil de México Federico Gómez/UNAM repository isolated from different sources and times were used. The DNA extraction was performed using an InstaGene™ Matrix (Bio-Rad, Hercules, CA, USA) commercial kit following the manufacturer’s instructions, and PCR products were compared with the UPEC CFT073 positive control strain.

#### 4.2.9. Analysis of *ompA* Sequences from *E. coli*

For the creation of a database for the diversity of the gene, 950 sequences of *ompA* from the GenBank were recovered and annotated. The sequences were aligned using the MUSCLE algorithm, the identity percentage was calculated, and a phylogenetic tree was constructed with the maximum similarity method using MEGA X software [51].

### 4.3. Statistical Analysis

Statistical analyses were computed using GraphPad Prism v. 6.00 (GraphPad Software, San Diego, CA, USA) software. A one-way ANOVA followed by Tukey’s test was used to analyze the results of the immunodetection ELISA assays, *p*-values of <0.05 were considered significant. A two-tailed Fisher’s exact test was used to analyze the results of the *ompA* gene prevalence in *E. coli* isolates, and *p*-values of <0.05 were considered significant.

## Figures and Tables

**Table 1:** Serotype prevalence of *E. coli* strains associated to recurrent urinary tract infections UTIs.

*E. coli* Serotypes ^‡^	No. of Urocultures *	No. of Patients *	No. of Strains per Serotype (%)
O25:H4	30	10	295 (41)
O75:HNM	15	4	150 (20.8)
O6:H1	4	2	40 (5.5)
O8:HNM; O20:H9; O22:H1 & O44:HNM	2	1	20 (2.8)
O29:H10; O73:H18; O75:H25; O101:H19; O152:H8; O174:H28 & O175:H25	1	1	10 (1.4)
O138:H6 & O32:HND	1	1	5 (0.7)
Prevalent serotype subtotal	-	-	645 (89.6)
OND:HNM	3	3	30 (4.1)
OND:HND	2	2	20 (2.8)
OND:H9 &OR:H18	1	1	10 (1.4)
OND:H21	1	1	5(0.7)
Non-serotypable strains subtotal	-	-	75 (10.4)
Total	-	-	720

^‡^ NM, non-motile; ND, non-determined; OR, rough phenotype.* In some cases, more than one *E. coli* serotype was isolated from the same uroculture.

**Table 2:** *ompA* prevalence in *E. coli* strains isolated from difference sources.

Source of *E. coli* Isolate	No. of Strains	ompA	* *p*-Value
		No. (%)	
Human feces	23	10 (43)	-
Cattle	45	26 (58)	0.3108
STEC, EAEC & DEC strains from human patients	10	7 (70)	0.2587
*E. coli* strains associated with UTIs	70	38 (54)	0.4720
**Total**	148	81 (55)	0.2617

* *p*-value of two-tailed Fisher’s exact test comparing human feces isolates.

## Supplementary Materials

The following are available online at https://www.mdpi.com/2076-0817/9/2/102/s1, Figure S1: *E. coli ompA* phylogenetic tree, Table S1: Patients follow up of the treatment with autologous BL, Table S2: Protein ID.

## References

[1] Flores-Mireles A.L., Walker J.N., Caparon M., Hultgren S.J. (2015). Urinary tract infections: Epidemiology, mechanisms of infection and treatment options. Nat. Rev. Microbiol..

[2] Foxman B. (2014). Urinary tract infection syndromes: Occurrence, recurrence, bacteriology, risk factors, and disease burden. Infect. Dis. Clin. N. Am..

[3] Foxman B. (2010). The epidemiology of urinary tract infection. Nat. Rev. Urol..

[4] O’Brien V.P., Hannan T.J., Nielsen H.V., Hultgren S.J. (2016). Drug and Vaccine Development for the Treatment and Prevention of Urinary Tract Infections. Microbiol. Spectr..

[5] Ochoa S.A., Cruz-Córdova A., Luna-Pineda V.M., Reyes-Grajeda J.P., Cázares-Domínguez V., Escalona G., Sepúlveda-González M.E., López-Montiel F., Arellano-Galindo J., López-Martínez B. (2016). Multidrug- and Extensively Drug-Resistant Uropathogenic Escherichia coli Clinical Strains: Phylogenetic Groups Widely Associated with Integrons Maintain High Genetic Diversity. Front. Microbiol..

[6] WHO Publishes List of Bacteria for Which New Antibiotics Are Urgently Needed. https://www.who.int/news-room/detail/27-02-2017-who-publishes-list-of-bacteria-for-which-new-antibiotics-are-urgently-needed.

[7] 20 Principales Nacional. http://epidemiologia.salud.gob.mx/anuario/html/principales_nacional.html.

[8] Stamm W.E., Norrby S.R. (2001). Urinary Tract Infections: Disease Panorama and Challenges. J. Infect. Dis..

[9] O’Brien V.P., Hannan T.J., Schaeffer A.J., Hultgren S.J. (2015). Are you experienced? Understanding bladder innate immunity in the context of recurrent urinary tract infection. Curr. Opin. Infect. Dis..

[10] Yang B., Foley S. (2018). First experience in the UK of treating women with recurrent urinary tract infections with the bacterial vaccine Uromune^®^. BJU Int..

[11] De Benedetto F., Sevieri G. (2013). Prevention of respiratory tract infections with bacterial lysate OM-85 bronchomunal in children and adults: A state of the art. Multidiscip. Respir. Med..

[12] Micali S., Isgro G., Bianchi G., Miceli N., Calapai G., Navarra M. (2014). Cranberry and recurrent cystitis: More than marketing?. Crit. Rev. Food Sci. Nutr..

[13] Storme O., Tirán Saucedo J., Garcia-Mora A., Dehesa-Dávila M., Naber K.G. (2019). Risk factors and predisposing conditions for urinary tract infection. Ther. Adv. Urol..

[14] Paniagua-Contreras G.L., Monroy-Pérez E., Rodríguez-Moctezuma J.R., Domínguez-Trejo P., Vaca-Paniagua F., Vaca S. (2017). Virulence factors, antibiotic resistance phenotypes and O-serogroups of Escherichia coli strains isolated from community-acquired urinary tract infection patients in Mexico. J. Microbiol. Immunol. Infect..

[15] Robino L., García-Fulgueiras V., Araujo L., Algorta G., Pírez M.C., Vignoli R. (2014). Urinary tract infection in Uruguayan children: Aetiology, antimicrobial resistance and uropathogenic Escherichia coli virulotyping. J. Glob. Antimicrob. Resist..

[16] Jadhav S., Hussain A., Devi S., Kumar A., Parveen S., Gandham N., Wieler L.H., Ewers C., Ahmed N. (2011). Virulence characteristics and genetic affinities of multiple drug resistant uropathogenic Escherichia coli from a semi urban locality in India. PLoS ONE.

[17] Molina-López J., Aparicio-Ozores G., Ribas-Aparicio R.M., Gavilanes-Parra S., Chávez-Berrocal M.E., Hernández-Castro R., Manjarrez-Hernández H.Á. (2011). Drug resistance, serotypes, and phylogenetic groups among uropathogenic Escherichia coli including O25-ST131 in Mexico City. J. Infect. Dev. Ctries..

[18] Morales-Espinosa R., Hernandez-Castro R., Delgado G., Mendez J.L., Navarro A., Manjarrez A., Cravioto A. (2016). UPEC strain characterization isolated from Mexican patients with recurrent urinary infections. J. Infect. Dev. Ctries..

[19] Belmont-Monroy L., Ribas-Aparicio R.M., Navarro-Ocaña A., Manjarrez-Hernández H.Á., Gavilanes-Parra S., Aparicio-Ozores G., Cauich-Sánchez P.I., Garza-Ramos U., Molina-López J. (2017). Characterization of Escherichia coli causing community acquired urinary tract infections in Mexico City. Diagn. Microbiol. Infect. Dis..

[20] Toval F., Köhler C.-D., Vogel U., Wagenlehner F., Mellmann A., Fruth A., Schmidt M.A., Karch H., Bielaszewska M., Dobrindt U. (2014). Characterization of Escherichia coli isolates from hospital inpatients or outpatients with urinary tract infection. J. Clin. Microbiol..

[21] Clermont O., Bonacorsi S., Bingen E. (2000). Rapid and Simple Determination of the Escherichia coli Phylogenetic Group. Appl. Environ. Microbiol..

[22] Vejborg R.M., Hancock V., Schembri M.A., Klemm P. (2011). Comparative Genomics of Escherichia coli Strains Causing Urinary Tract Infections. Appl. Environ. Microbiol..

[23] Taha Neto K.A., Nogueira Castilho L., Reis L.O. (2016). Oral vaccine (OM-89) in the recurrent urinary tract infection prophylaxis: A realistic systematic review with meta-analysis. Actas Urol. Esp..

[24] Brumbaugh A.R., Mobley H.L.T. (2012). Preventing urinary tract infection: Progress toward an effective Escherichia coli vaccine. Expert Rev. Vaccines.

[25] Meredith M., Chiavaroli C., Bauer H.G. (2009). Immunotherapy for Recurrent Urinary Tract Infections: Effects of an Escherichia coli Extract. Curr. Urol..

[26] Wills-Karp M., Karp C.L. (2004). Chitin checking—Novel insights into asthma. N. Engl. J. Med..

[27] Bessler W.G., Puce K., vor dem Esche U., Kirschning C., Huber M. (2009). Immunomodulating effects of OM-89, a bacterial extract from Escherichia coli, in murine and human leukocytes. Arzneimittelforschung.

[28] Lorenzo-Gómez M.F., Padilla-Fernández B., García-Cenador M.B., Virseda-Rodríguez Á.J., Martín-García I., Sánchez-Escudero A., Vicente-Arroyo M.J., Mirón-Canelo J.A. (2015). Comparison of sublingual therapeutic vaccine with antibiotics for the prophylaxis of recurrent urinary tract infections. Front. Cell. Infect. Microbiol..

[29] Abraham S.N., Miao Y. (2015). The nature of immune responses to urinary tract infections. Nat. Rev. Immunol..

[30] Purves J.T., Hughes F.M. (2016). Inflammasomes in the urinary tract: A disease-based review. Am. J. Physiol. Renal Physiol..

[31] Huber M., Krauter K., Winkelmann G., Bauer H.W., Rahlfs V.W., Lauener P.A., Blessmann G.S., Bessler W.G. (2000). Immunostimulation by bacterial components: II. Efficacy studies and meta-analysis of the bacterial extract OM-89. Int. J. Immunopharmacol..

[32] Forde B.M., Roberts L.W., Phan M.-D., Peters K.M., Fleming B.A., Russell C.W., Lenherr S.M., Myers J.B., Barker A.P., Fisher M.A. (2019). Population dynamics of an Escherichia coli ST131 lineage during recurrent urinary tract infection. Nat. Commun..

[33] Sarowska J., Futoma-Koloch B., Jama-Kmiecik A., Frej-Madrzak M., Ksiazczyk M., Bugla-Ploskonska G., Choroszy-Krol I. (2019). Virulence factors, prevalence and potential transmission of extraintestinal pathogenic Escherichia coli isolated from different sources: Recent reports. Gut Pathog..

[34] Bäckhed F., Normark S., Schweda E.K.H., Oscarson S., Richter-Dahlfors A. (2003). Structural requirements for TLR4-mediated LPS signalling: A biological role for LPS modifications. Microbes Infect..

[35] Trent M.S., Stead C.M., Tran A.X., Hankins J.V. (2006). Diversity of endotoxin and its impact on pathogenesis. J. Endotoxin Res..

[36] Poxton I.R. (1995). Antibodies to lipopolysaccharide. J. Immunol. Methods.

[37] Smith S.G.J., Mahon V., Lambert M.A., Fagan R.P. (2007). A molecular Swiss army knife: OmpA structure, function and expression. FEMS Microbiol. Lett..

[38] Alteri C.J., Mobley H.L.T. (2009). Quantitative Profile of the Uropathogenic Escherichia coli Outer Membrane Proteome during Growth in Human Urine. Infect. Immun..

[39] Nicholson T.F., Watts K.M., Hunstad D.A. (2009). OmpA of Uropathogenic Escherichia coli Promotes Postinvasion Pathogenesis of Cystitis. Infect. Immun..

[40] Wu H.-H., Yang Y.-Y., Hsieh W.-S., Lee C.-H., Leu S.-J.C., Chen M.-R. (2009). OmpA Is the Critical Component for Escherichia coli Invasion-Induced Astrocyte Activation. J. Neuropathol. Exp. Neurol..

[41] Da Silva L.C., de Mello Santos A.C., Silva R.M. (2017). Uropathogenic Escherichia coli pathogenicity islands and other ExPEC virulence genes may contribute to the genome variability of enteroinvasive E. coli. BMC Microbiol..

[42] Cordeiro M.A., Werle C.H., Milanez G.P., Yano T. (2016). Curli fimbria: An Escherichia coli adhesin associated with human cystitis. Braz. J. Microbiol..

[43] Osman K.M., Kappell A.D., ElHofy F., Orabi A., Mubarak A.S., Dawoud T.M., Moussa I.M., Hessain A.M. (2018). Urinary tract infection attributed to Escherichia coli isolated from participants attending an unorganized gathering. Future Microbiol..

[44] Liao C., Liang X., Yang F., Soupir M.L., Howe A.C., Thompson M.L., Jarboe L.R. (2017). Allelic Variation in Outer Membrane Protein A and Its Influence on Attachment of Escherichia coli to Corn Stover. Front. Microbiol..

[45] Camprubí-Font C., Ruiz del Castillo B., Barrabés S., Martínez-Martínez L., Martinez-Medina M. (2019). Amino Acid Substitutions and Differential Gene Expression of Outer Membrane Proteins in Adherent-Invasive Escherichia coli. Front. Microbiol..

[46] Wilson M.L., Gaido L. (2004). Laboratory diagnosis of urinary tract infections in adult patients. Clin. Infect. Dis. Off. Publ. Infect. Dis. Soc. Am..

[47] Ørskov F., Ørskov I., Jann K., Jann B. (1990). The Serology of Capsular Antigens. Bacterial Capsules.

[48] Molloy M.P. (2008). Isolation of bacterial cell membranes proteins using carbonate extraction. Methods Mol. Biol. Clifton NJ.

[49] Mazariego-Espinosa K., Cruz A., Ledesma M.A., Ochoa S.A., Xicohtencatl-Cortes J. (2010). Longus, a type IV pilus of enterotoxigenic Escherichia coli, is involved in adherence to intestinal epithelial cells. J. Bacteriol..

[50] Ewers C., Li G., Wilking H., Kiessling S., Alt K., Antáo E.-M., Laturnus C., Diehl I., Glodde S., Homeier T. (2007). Avian pathogenic, uropathogenic, and newborn meningitis-causing Escherichia coli: How closely related are they?. Int. J. Med. Microbiol. IJMM.

[51] Kumar S., Stecher G., Li M., Knyaz C., Tamura K. (2018). MEGA X: Molecular Evolutionary Genetics Analysis across Computing Platforms. Mol. Biol. Evol..

