# Supplementary material for: Effect and Analysis of Bacterial Lysates for the Treatment of Recurrent Urinary Tract Infections in Adults
Source: Pathogens. 2020 Feb 6;9(2):102. doi: 10.3390/pathogens9020102 (PMC7168004; doi:10.3390/pathogens9020102)
Supplement: Supplementary file 1 [file pathogens-09-00102-s001.zip › Figure S1..pptx]

## Slide 1
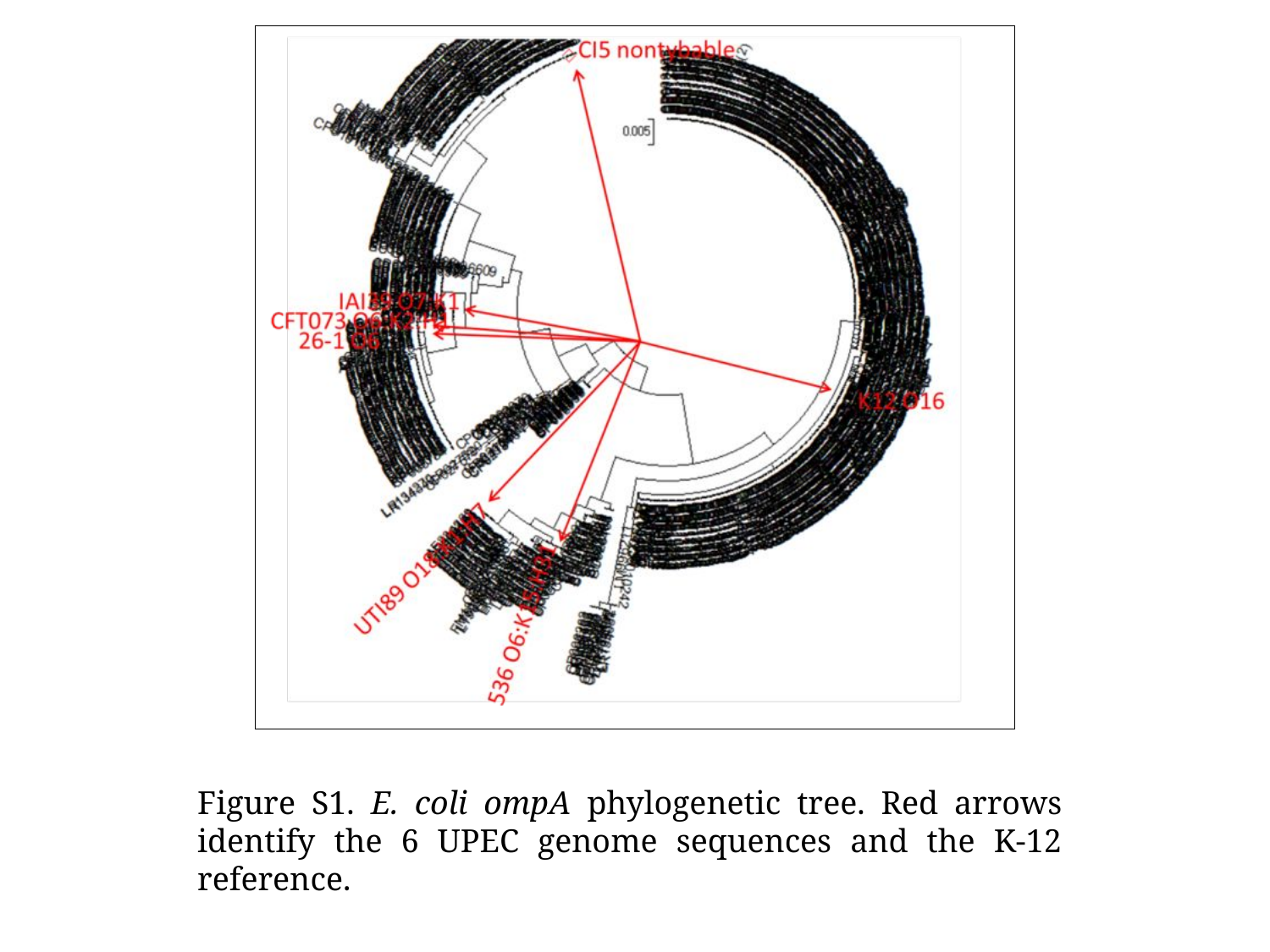

Figure S1. E. coli ompA phylogenetic tree. Red arrows identify the 6 UPEC genome sequences and the K-12 reference.
